# De novo synthesis of nervonic acid and optimization of metabolic regulation by *Yarrowia lipolytica*

**DOI:** 10.1186/s40643-023-00689-6

**Published:** 2023-10-06

**Authors:** Xin-Ru Zhao, Xin-Liang Chen, Jing-Lin Yang, Qi Gao, Jiang-Ting Shi, Qiang Hua, Liu-Jing Wei

**Affiliations:** 1 State Key Laboratory of Bioreactor Engineering, East China University of Science and Technology, 130 Meilong Road, Shanghai, 200237 People’s Republic of China; 2 Shanghai Collaborative Innovation Center for Biomanufacturing Technology, 130 Meilong Road, Shanghai, 200237 China

**Keywords:** Nervonic acid, *Yarrowia lipolytica*, De novo, Metabolic engineering, Colleseed oil

## Abstract

Nervonic acid, a natural fatty acid compound and also a core component of nerve fibers and nerve cells, has been widely used to prevent and treat related diseases of the brain nervous system. At present, fatty acids and their derivatives are mainly obtained by natural extraction or chemical synthesis which are limited by natural resources and production costs. In this study, the de novo synthetic pathway of nervonic acid was constructed in *Yarrowia lipolytica* by means of synthetic biology, and the yield of nervonic acid was further improved by metabolic engineering and fermentation optimization. Specially, heterologous elongases and desaturases derived from different organism were successfully expressed and evaluated for their potential for the production of nervonic acid in *Y. lipolytica*. Meanwhile, we overexpressed the genes involved in the lipid metabolism to increase the nervonic acid titer to 111.6 mg/L. In addition, the potential of adding oil as auxiliary carbon sources for nervonic acid production by the engineered *Y. lipolytica* was analyzed. The results indicated that supplementation with colleseed oil as an auxiliary carbon source can be beneficial for the nervonic acid productivity, which led to the highest concentration of 185.0 mg/L in this work. To summarize, this study describes that the *Y. lipolytica* can be used as a promising platform for the production of nervonic acid and other very long-chain fatty acids.

**Graphical Abstract:**

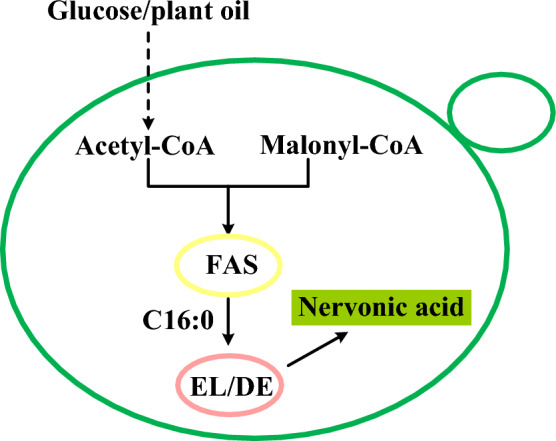

**Supplementary Information:**

The online version contains supplementary material available at 10.1186/s40643-023-00689-6.

## Introduction

Nervonic acid is a natural fatty acid compound and also a core component of nerve fibers and nerve cells. As a necessary fatty acid, nervonic acid is essential for brain development and maintenance of neuronal biosynthesis and improvement. It can be used for the prevention and treatment of diseases related to the brain nervous system, such as mental disorders and cognitive disorders (Tanaka et al. 2007). Studies have shown that nervonic acid can inhibit HIV-1 RT activity in a dose-dependent manner as a non-competitive inhibitor (Kasai et al. 2002). Reduced levels of nervonic acid in individuals are strongly associated with a higher risk of psychiatric disorders, and therefore, several neurological disorders, such as demyelinating diseases, can be treated with nervonic acid supplementation (Amminger et al. 2012; Raoul and Coupland 2001; Vozella et al. 2017). Nervonic acid is also a natural component of breast milk, which helps in the development of the infant’s nervous system and promotes their growth. It has been demonstrated that increasing the amount of nervonic acid in the daily diet of mice improves their energy metabolism, which may be an effective strategy for the treatment of obesity and obesity complications (Kepple et al. 2020). Nervonic acid has a valuable biological function, which makes it play an important role in pharmacological and nutritional applications (Li et al. 2019). Currently, nervonic acid is mainly obtained by extraction from plant tissues or chemical synthesis. Each of these extraction methods has different limitations. Chemical synthesis of nervonic acid has low yield and many by-products (Rongkai et al. 2018). The extraction of nervonic acid from plants is the most commonly used method, and this method has some limitations because of the limitations of the plant growth cycle and climatic conditions (Tang et al. 2013). Therefore, it is time to explore a green and feasible way to biosynthesizing nervonic acid.

In recent years, research on the synthesis of fatty acids using microorganisms has made great progress (Rongkai et al. 2018). Developments in synthetic biology and metabolic engineering have greatly facilitated the manipulation of microbial metabolic pathways and have contributed significantly to the production of a wide range of chemicals (Li et al. 2019). For example, the filamentous fungi *Mortierella capitata RD000969* isolated from soil can accumulate nervonic acid for 6.94% of the total fatty acid (Umemoto et al. 2014). In *Saccharomyces cerevisiae*, β-estradiol inducible expression system (EIES) was used to enhance the intracellular production of nervonic acid. Then the level of nervonic acid was further increased by overexpression of *KCS* and *ELOVL1* genes and knockout of *ELO2* (Liu et al. 2020). It has been reported that the production of nervonic acid in *Rhodosporidium toruloides* has been achieved by screening and expressing elongation genes (3-ketoacyl-CoA synthases, KCS) from different plant sources (Fillet et al. 2017). This study has proved that the copy number of *KCS* gene and the push/pull strategy for *KCS* gene preference increased the contents of C24:1 and C22:1 fatty acids. By optimizing the fermentation conditions, the yield of nervonic acid in the 7 L bioreactor reached 20–30% of the yield of very long-chain fatty acids (VLCFAs).

*Yarrowia lipolytica*, as a GRAS grade yeast strain, is one of the most studied “unconventional” yeast species (Bourdichon et al. 2012). This oleaginous yeast is an attractive biorefinery platform strain for industrial-scale production of oleochemicals due to its robustness, natively high flux toward fatty acid biosynthesis and tolerance toward harsh fermentation conditions (Beopoulos et al. 2009). The genome of *Y. lipolytica* has been sequenced and gene-editing tools developed and used are becoming more sophisticated (Liu and Alper 2014). *Y. lipolytica* has a large intercellular pool size of acetyl-CoA (the fatty acid backbone precursor), which can be generated through various metabolic pathways, such as citrate degradation catalyzed by ATP citrate lyase (ACL), fatty acids through β-oxidative degradation, and conversion of acetic by acetyl-CoA synthase. Under nitrogen restriction, mitochondria secrete citric acid which produces acetyl-CoA in the presence of ACL and then forms malonyl-CoA catalyzed by acetyl-CoA carboxylase (ACC1). Acyl-CoA was used as the starting point and malonyl-CoA as the extension unit to generate 16 and 18 chain lengths acyl-CoA. Further extended and desaturated with 16:0 and 18:0 activated molecules as the precursor to obtain fatty acids with various chain lengths and saturation via Kennedy pathway (Beopoulos et al. 2009). Each elongation consumes two molecules of NADPH, of which there are two pathways for the source of NADPH: One is through the decarboxylation reaction catalyzed malate dehydrogenase in the cytoplasm and the other is pentose phosphate pathway (Wasylenko et al. 2015). Therefore, the oleaginous yeast *Y. lipolytica* is particularly expected to be a metabolic engineering platform strain for nervonic acid synthesis.

During the preparation of this manuscript, Wang et al. engineered *Y. lipolytica* to produce up to 57.48 g/L of microbial oil with 23.44% nervonic acid in fed-batch fermentation, the highest production titer so far described in *Y. lipolytica*. The authors combined orthogonal plant and non-plant fatty acid biosynthesis pathways in *Y. lipolytica*, used a “block-pull-restrain” strategy to increase precursor production, and strengthened Triacylglycerols (TAGs) synthesis to improve lipid pool (Wang et al. 2023).

**Fig. 1:**
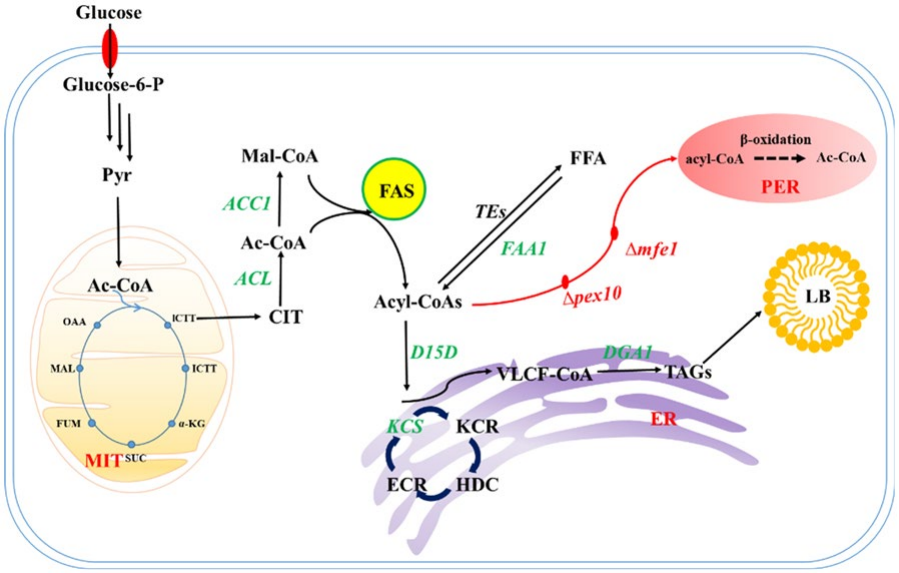
Biosynthesis pathway for nervonic acid production in the yeast *Y. lipolytica*. Nervonic acid is biosynthesized from the initiation unit of acetyl-CoA and the extension unit of malonyl-CoA with fatty acid synthetase. Green words represent the heterogeneous expression pathways; black words represent the native pathways; and red words represent selected for disruption in this study. Pyr: pyruvic acid; Ac-CoA: acetyl coenzyme A; CIT: citric acid; Mal-CoA: Malonyl coenzyme A; FAS: fatty acid synthase; TE: thioesterase; ACL: ATP citrate lyase; FAA1: acyl-CoA synthetase; FFA: free fatty acid; KS: ketoacyl-CoA synthase; KR: 3-ketoacyl-CoAreductase; DH: 3-hydroxyacyl-CoA dehydratase; ER: enoyl-CoA reductase; D15D: ∆15 desaturase; TAG: triacylglycerol

In this work, we constructed the de novo synthesis of nervonic acid in oleaginous yeast *Y. lipolytica* (Fig. 1). In order to further increase the production of nervonic acid, the elongation genes and desaturation genes in the process of nervonic acid synthesis were screened and overexpressed in *Y. lipolytica*. Meanwhile, the expression patterns of different combinations of key genes were explored to further enhance the production of nervonic acid. Moreover, we analyzed the potential of different auxiliary carbon sources for the production of nervonic acid by *Y. lipolytica* and first found that colleseed oil as auxiliary carbon source was helpful to increase nervonic acid production.

## Materials and methods

### Plasmids, strains, and medium

*Yarrowia lipolytica* strain ATCC MYA2613 (Po1f) was used as the initial strain of the engineered strains. Construction and amplification of plasmids were dependent on *E. coli* strain JM109, which was cultured in Luria–Bertani (LB) medium and grew at 37 °C. LB medium (10 g/L yeast extract, 20 g/L peptone, 10 g/L NaCl, and 15 g/L Bacto agar) was added with different resistance to construct plasmids such as 50 mg/L of kanamycin and 100 mg/L of ampicillin. The *Y. lipolytica* strains were cultivated at 30 ℃ in YPD medium (10 g/L yeast extract, 20 g/L peptone, 20 g/L glucose, and 15 g/L Bacto agar). The YNB medium, which contains 6.7 g/L yeast nitrogen base without amino acids, 20 g/L glucose and 15 g/L Bacto agar, was used to screen transformants by adding 100 mg/L leucine or uracil. In this study, 5-fluoroorotic acid (1 g/L of 5-FOA) was added to YPD medium for the recovery of URA3 screening markers. All strains constructed and used in this study are listed in Table 1.

**Table 1:** Strains used in this study

Strains	Descriptions	Source
*E. coli* JM109	recA1, endA1, gyrA96, thi, hsdR17, supE44, relA1, Δ(lac-proAB)/F[traD36, proab^+^, lacI^q^, lacZΔM15]	Invitrogen
*Y*. *lipolytica* Po1f	MatA, leu2-270, ura3-302, xpr2-322, axp1-2	Nicaud (2012)
GQY-∆PEX10	Po1f-∆PEX10	Gao et al. (2018)
GQ06	Po1f-∆PEX10 integrated MaELO3 by CRISPR/Cas9 at F1 site	Gao et al. (2020)
NA01	GQ06 integrated optimized AtKCS by CRISPR/Cas9 at A3 site	This study
NA02	NA01 integrated optimized CraKCS by CRISPR/Cas9 at F1-3 site	This study
NA03	NA01 integrated optimized CgKCS by CRISPR/Cas9 at AXP site	This study
NA04	NA03 integrated optimized CraKCS by CRISPR/Cas9 at F1-3 site	This study
NA05	NA04 cells harboring pINA1312-P_UT_- MaD15D	This study
NA06	NA04 cells harboring pINA1312-P_UT_- CsD15D	This study
NA07	NA04 integrated optimized CgKCS-L-MaD15D by CRISPR/Cas9 at A1-2 site	This study
NA08	NA07 integrated optimized CgKCS-L-MaD15D by CRISPR/Cas9 at E1-3 site	This study
NA09	NA04 cells harboring pINA1312-P_UT_- DGA1	This study
NA10	NA08 cells harboring pINA1312-P_UT_- OLE1	This study
NA11	NA08 cells harboring pINA1312-P_UT_- DGA1-L-OLE1	This study
NA12	NA08 cells harboring pINA1312-P_UT_- OLE1-L-DGA1	This study
NA13	NA12 cells harboring pINA1269- OLE1-L-DGA1	This study
NA14	NA10 cells harboring pINA1269-DGA1	This study
NA15	NA12 cells harboring pINA1269-DGA1	This study
NA16	NA12 cells harboring pINA1269-MaELO3	This study
NA17	NA12 cells harboring pINA1269-MaELO3-AtKCS	This study
NA18	NA12 cells harboring pINA1269-MaELO3-CraKCS	This study
NA19	NA12 cells harboring pINA1269-CgKCS	This study
NA20	NA04 cells harboring pINA1269-ACL	This study
NA21	NA04 cells harboring pINA1269-ACS2	This study
NA22	NA04 cells harboring pINA1269-ACC1	This study
NA23	NA20 cells harboring pINA1312-ACS2	This study
NA24	NA04 integrated FAA1 by CRISPR/Cas9 at MFE site	This study

### Construction of plasmids and yeast transformation

In this study, two integrative plasmids, pINA1312 and pINA1269, and CRISRPR/Cas9 system were used for metabolic engineering modification of the strains. All constructed strains are shown in Table 1. The elongation enzyme gene (*CgKCS*) from *Cardamine graeca* and the ∆15 desaturase genes (*MaD15D/CsD15D*) from *Mortierella alpine* and *Cannabis sativa* were synthesized and codon optimized. Primers were designed to amplify target genes by PCR, and the amplified genes were linked to plasmids pINA1312 or pINA1269 that had been digested by the ClonExpress® II One Step Cloning Kit (Vazyme Biotech, Nanjing, China). Then the recombinant plasmid carrying target gene was obtained. The primers involved in this study are all shown in Additional file 1: Table S1. Promoters with different strengths were constructed to improve the efficiency of gene expression. After construction of the recombinant plasmid, it was linearized by the corresponding enzyme and then transferred into yeast cells by Frozen-EZ yeast transformation II Kit (Zymo Research, Irvine, CA).

The CRISPR/Cas9 system is able to knock out the gene and knock in the target gene at the same time. Taking the *CgKCS* gene which was inserted into the AXP site as an example, primers with 20 bp homologous sequences at both ends of insertion site were used to obtain the gene fragment of *CgKCS* by PCR. Then plasmid pHR_AXP_hrGFP digested with *SpeI* and *AvrII* connected with gene fragment of *CgKCS* to obtain recombinant plasmid pHR_AXP_CgKCS. Finally, the single gRNA and recombinant plasmid pHR_AXP_CgKCS were transformed into corresponding yeast cell together. All the primers used and plasmids constructed are shown in Additional file 1: Tables S2, S3.

### Growth condition and auxiliary carbon source

*Yarrowia lipolytica* strains were cultured in 2 mL YPD at 30 °C (220 rpm) and then inoculated in 250 mL triangular flask containing 50 mL YPD with an initial OD_600_ of 0.01. The strains were cultured for 72 h under the same conditions. 0.25 mL of different carbon sources (ω-9 octadecanoic acid, soybean oil, colleseed oil, sunflower seed oil, waste cooking oil) were added to 50 mL YPD. On this basis, gradient experiments of colleseed oil supplemental levels were designed, such as 0, 0.25, 0.5, 0.75, 1.0, and 1.25 mL were added to 50 mL YPD.

### Extraction of VLCFAs

For the analysis of VLCFAs, the lipids were extracted and transmethylated into FAMEs. Detailed method was given in previous work (Nambou et al. 2014). 20 mL of the fermentation medium was taken into a 50 mL centrifuge tube and centrifuged at 6000 rpm for 5 min. The supernatant was discarded, and then 15 mL of ddH_2_O was added to the centrifuge tube. The mixture was thoroughly mixed and subjected to centrifugation under the same conditions. After repeating the above procedure, 5 mL 4 M HCl was added to the collected cells. The mixture was oscillated and then held for 30 min at 37 ℃ at 220 rpm. Next, the test tube was kept in boiling water bath and ice for 5 min, and the operation was repeated again. Then 20 mL of methanol and chloroform mixed solution was added into the test tube, in which the volume ratio of methanol to chloroform was 1:2. After 30 min at 37 °C, the underlying liquid was centrifuged (4800 rpm, 5 min) and then sucked into a glass tube and dried in an oven at 105 °C. After about 12 h, the test tube was taken out and then 3 mL of 0.5 mol/L methanol potassium hydroxide solution was added into the test tube when the test tube is restored to room temperature. Ultrasound was used to dissolve the oil in the tube, and the tube was kept in a water bath at 75 ℃ for 20 min. 3 mL of 14% boron trichloride solution was added to the test tube and the same condition was kept for 20 min. Then the tube was taken out, and 1 mL saturated NaCl and 0.5 mL *n*-hexane were added to it. The mixture was thoroughly mixed and the upper solution was centrifuged at 12,000 rpm for 2 min. Then the upper liquid was diluted and it was mixed with the internal standard at a volume ratio of 1:4 to get the sample to be tested.

### Gas chromatography coupled with mass spectrometry (GC−MS) analysis of VLCFAs

The sample was analyzed by GC–MS which was carried out using an Agilent System 6890 gas chromatograph (GC) with an Agilent 5975 quadrupole mass selective detector (MSD) equipped with a HP-5 column (30 m × 0.25 mm × 0.25 μm, Agilent, Santa Clara, CA, USA). The initial temperature of GC was held at 150 °C for 2 min and then at a rate of 20 °C/min to 180 °C. And then it went up to 200 °C at a rate of 8 °C/min. Then in 18 min the temperature reached 218 °C, raised to 250 °C at 8 °C/min. The temperature subsequently raised to 300 °C in 3.4 min. The split ratio was 20:1. The quantitative analysis was carried out by the corresponding fatty acid methyl ester standards.

## Results and discussion

### De novo synthesis of nervonic acid in *Y. lipolytica*

**Fig. 2:**
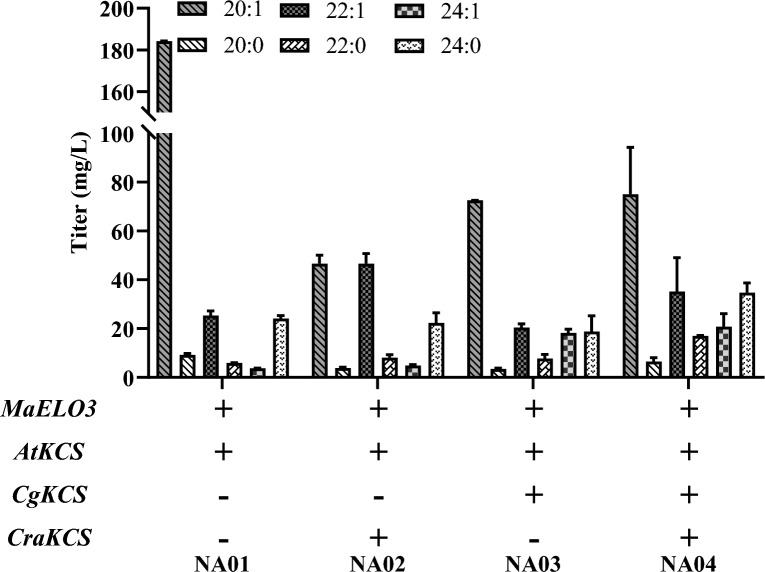
Effects of overexpression of elongation genes from different sources on nervonic acid production on solid medium (YPD). The data show the average of two independent experiments, with the error bars representing standard deviations. *MaELO3*: *Mortierella alpina ELO3* gene; *AtKCS*: *Arabidopsis thaliana KCS* gene; *CgKCS*: *Cardamine graeca KCS* gene; *CraKCS*: *Crambe abyssinica KCS* gene

We previously engineered *Y. lipolytica* to produce VLCFAs with carbon chain lengths up to 24 by co-expression heterologous C16/18-elongase from *Mortierella alpina* (MaELO3), β-ketoacyl-CoA synthases (KCSs) from *Arabidopsis thaliana* (AtKCS), and *Crambe abyssinica* (CraKCS) combining with the deletion of *PEX1* (Gao et al. 2020). Although VLCFAs metabolism was successfully engineered, the resulting strain GQ07 only accumulates marginal nervonic acid (C24:1), and the titer needs to be further improved. Owing to the limitation of auxotrophic markers of plasmids, here, we re-engineered VLCFAs metabolism pathway into chromosome using the recently established CRISPR/Cas9 technology without the selection marker (Schwartz et al. 2016). The use of hybrid promoter UAS4B-TEF (UT) provided an excellent platform for high gene expression in *Y. lipolytica*. Therefore, we used this for the overexpression of the codon-optimized *MaELO3*, *AtKCS*, and *CraKCS* genes at the F1, A3, and F1–3 integration sites of *Y. lipolytica* GQY-∆PEX10 strain (Zhang et al. 2020). Previous study confirmed that KCS enzyme from *C. graeca* has the ability to elongate erucoyl-CoA (C22:1-CoA) to nervonic acid by in vitro activity assays (Taylor et al. 2009). In another work, heterologous expression of *KCS* gene from *C. graeca* in *R. toruloides* efficiently catalyzed all elongation steps to produce nervonic acid (Fillet et al. 2017). To elucidate the effects of *CgKCS* overexpression on nervonic acid production in *Y. lipolytica*, the codon-optimized *CgKCS* was integrated into the AXP site by CRISPR/Cas9 technology in the *MaELO3*, *AtKCS*-expressed background strain (NA01), yielding strain (NA03). As shown in Fig. 2, strain NA03 can produce about 18.2 mg/L of nervonic acid, which is approximately fourfold than that of strain NA01. These results clearly showed that the chain length of VLCFAs could be selectively modulated by engineering different sources of *KCS*. Consisting with previous reports, *CgKCS* gene could efficiently push elongation of the erucoyl-CoA pool to nervonic acid (Fillet et al. 2017). Strain NA04 obtained by simultaneous overexpression *MaELO3*, *AtKCS*, *CraKCS,* and *CgKCS* genes could produce 20.8 mg/L nervonic acid and this strain can be used as a host strain to further enhance the production of nervonic acid.

### Explore desaturase of nervonic acid synthesis

**Fig. 3:**
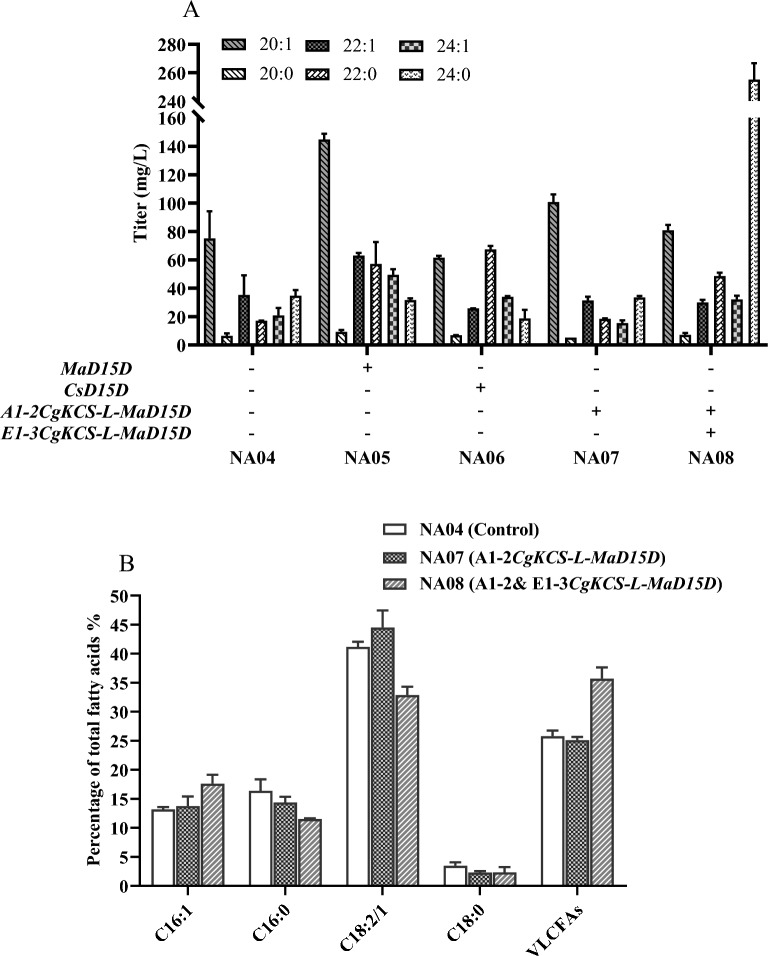
Heterologous desaturase expression in *Y. lipolytica*. **A** Screening the vary version of ∆15 desaturase and elongase for nervonic acid production. *MaD15D*: *Mortierella alpine* ∆15 desaturase gene; *CsD15D*: *Cannabis sativa* ∆15 desaturase gene. **B** Percentage of FA distribution in the engineered *Y. lipolytica* strains NA04 and NA08. The data are the averages of two biological replicates with error bars representing standard deviations

The fatty acid profile of the engineered *Y. lipolytica* NA04 strains revealed that the rewritten the elongation pathway can improve the accumulation of nervonic acid. However, engineered cells also produced high amounts of C24:0 saturated fatty acid (lignoceric acid), indicating that the desaturation from lignoceric acid to nervonic acid was the rate limiting step. Therefore, we speculated that introduction of heterologous desaturation pathways would further enhance nervonic acid production. Nervonic acid is produced from lignoceric acid catalyzed by the ∆-15 desaturase (D15D) enzyme. Several D15D enzymes have been identified until now, among which we selected two D15D enzymes from *M. alpina* (MaD15D) and *C. sativa* (CsD15D) for expression and characterization in *Y. lipolytica* NA04 strain under the control of hybrid promoter UAS4B-TEF (UT) using plasmid pINA1312 (Bielecka et al. 2014; Wang et al. 2011)*.* To ensure efficient expression of the *D15D*, the gene sequences were codon optimized for expression in *Y. lipolytica*. As shown in Fig. 3, CsD15D gave the less effect on the nervonic acid titer, while MaD15D gave the better performance on production of nervonic acid with a titer of 49.4 mg/L, which was 2.4-fold increase. These results illustrated that both of the elongation pathway and desaturation pathway are important for the biosynthesis of nervonic acid in *Y. lipolytica*.

To optimize the *KCS* and *D15D* expression, and release the auxotrophic markers as well, we tried to fuse *CgKCS* and *MaD15D* with a (GSG) linker between *CgKCS* and *MaD15D* (*CgKCS-L-MaD15D*) in the chromosome of *Y. lipolytica* NA07 strain using established CRISPR/Cas9 technology. However, when one copy of *CgKCS-L-MaD15D* was introduced into the A1-2 site of *Y. lipolytica* NA07 strain (Zhang et al. 2020), there was no enhancement of the production of nervonic acid. The reason may be due to the low expression of the fusion. As such, an extra copy of *CgKCS-L-MaD15D* was introduced into the E1-3 site of *Y. lipolytica* NA07 strain, resulting strain NA08 (Zhang et al. 2020). As shown in Fig. 3A, increasing the fusion copy of *CgKCS-L-MaD15D* in strain NA04 significantly enhanced the production of nervonic acid to 32.1 mg/L in shake flask culture. At the meantime, the amount of lignoceric acid produced by NA08 was 255.1 mg/L, which was 7.3-fold than that for control strain NA04. The FA profiles of the new engineered strain and the control strain were compared. The strain NA08 was found to synthesize more VLCFA (C20-C24) than the control strain NA04, while the C18:2/1 fatty acid content was reduced (Fig. 3B).

### Overexpression of genes *OLE1* and *DGA1* leads to significant increases in nervonic acid accumulation

**Fig. 4:**
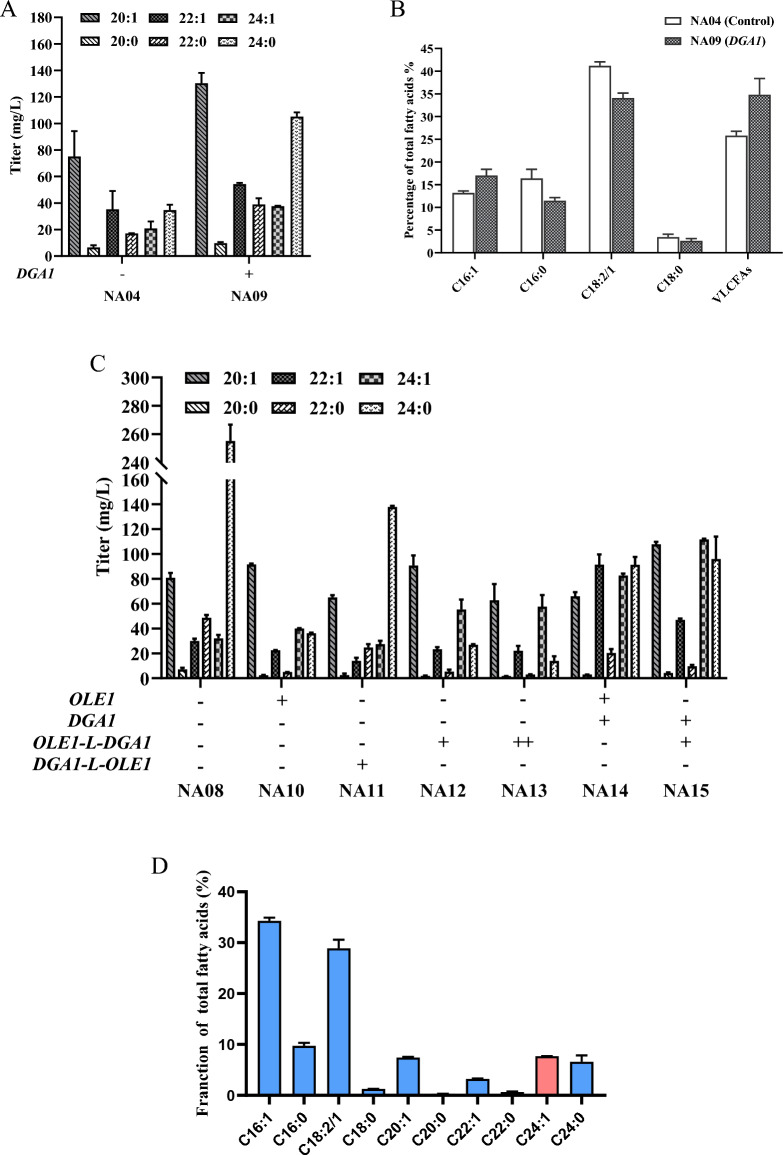
Effects of overexpression of genes *OLE1* and *DGA1* for the biosynthesis of nervonic acid in *Y. lipolytica*. **A** Overexpression of *DGA1* gene to improve nervonic acid production. **B** Percentage of FA distribution in the engineered *Y. lipolytica* strains NA04 and NA09. **C** Different combinations of *DGA1* and *OLE1* to increase nervonic acid production. **D** Percentage of FA distribution in the engineered *Y. lipolytica* strains NA15. The data are the averages of two biological replicates with error bars representing standard deviations

Diacylglycerol-acyltransferase (DGAT) catalyzes the acylation of diacylglycerol using acyl-CoA as the acyl donor. This enzyme has been postulated to be a main enzyme in boosting lipogenesis because it catalyzes the last step in TAG synthesis (Blazeck et al. 2014; Gajdos et al. 2016; Tai and Stephanopoulos 2013). The integrative vector pINA1312 carrying the *DGA1* gene under the control of hybrid promoter UAS4B-TEF (UT) was successfully integrated into the chromosome of NA04 strain. After 96 h cultivation, strain NA09, overexpressing the gene *DGA1*, significantly increased the level of nervonic acid production by 1.8-fold over strain NA04 (Fig. 4A). The distribution of fatty acids in strains NA04 and NA09 differed considerably in their percentage share. The C16:0 and C18:1/2 contents were drastically reduced and the VLCFAs content was increased in strain NA09 (Fig. 4B). Therefore, the target gene *DGA1* was selected for subsequent genetic modification.

*OLE1* of *Y. lipolytica* encodes the sole and essential ∆-9 stearoyl-CoA desaturase catalyzing the conversion of saturated to unsaturated fatty acids. Previous studies have shown that *OLE1* is important for lipogenesis (Flowers and Ntambi 2008; Qiao et al. 2015). Therefore, *OLE1* serves as an attractive engineering target to overproduce nervonic acid. We overexpressed the *OLE1* in the *Y. lipolytica* NA08 strain by introducing a native copy of the *OLE1* gene through integrated plasmid pINA1312 under the control of strong promoter UT resulting stain NA10. As shown in Additional file 1: Fig. S1, overexpression of *OLE1* increased the level of nervonic acid by 24.4% compared to the control strain NA08. Acetyl-CoA is a critical metabolite in carbon and energy metabolism and is involved in a variety of key metabolic functions (Gao et al. 2018; Huang et al. 2018). Here, we investigated the effect of overexpression of the key genes of acetyl-CoA metabolic pathway on the production of nervonic acid in *Y. lipolytica*. The *ACL* encoding the ATP-dependent citrate lyase, the *ACC1* encoding the acetyl-CoA carboxylase from *Y. lipolytica*, and *ACS2* encoding the acetyl-CoA synthetase gene from *S. cerevisiae* were overexpressed in the background strain through integrated plasmid pINA1269. Although there was no obviously difference in nervonic acid production between the engineered strain, the overexpression of *ACC1* resulted in a fourfold higher C24:0 titer than the control strain NA04 (Additional file 1: Fig. S2). We wanted to evaluate whether increasing the supply of the precursor acetyl-CoA level could increase nervonic acid production. *FAA1*, encoding acetyl-CoA synthetase, converts free fatty acids into acetyl-CoA, which enters the extended fatty acid cycle. The gene *MFE* encodes one of the enzymes involving in β-oxidation, which was often disrupted when engineering high lipid accumulation. To find out potential strategy promotes the biosynthesis of nervonic acid, *FAA1* overexpression cassette was inserted into *MFE* logic by CRISPR/Cas9 system resulting in strain NA22. The strain NA22 produced 28.3 mg/L nervonic acid in shake flasks, which was 1.36-fold higher than that of the control strain NA04 (Additional file 1: Fig. S3). This strategy might be a potential way to improve nervonic acid production in *Y. lipolytica*.

Since the single overexpression of *DGA1* or *OLE1* boosted the titer of nervonic acid in flask culture, we then reasoned that simultaneous co-overexpression of *DGA1* and *OLE1* would further increase nervonic acid accumulation. And we also performed a fusion strategy to evaluate whether fusion expression of the two enzymes could improve the production level of nervonic acid. *DGA1* and *OLE1* were fused with an artificial flexible linker (GSG) as either *DGA1-L-OLE1* or *OLE1-L-DGA1*, but only the *OLE1-L-DGA1* fusion resulted in a 1.7-fold increase in nervonic acid in engineered *Y. lipolytica* NA08 (Fig. 4C). We also tried overexpression *DGA1*, *OLE1,* and *OLE1-L-DGA1* and obtained three different strains. In comparison, strain NA15, which was simultaneously overexpressed *DGA1*and *OLE1-L-DGA1*, had the highest yield of nervonic acid (111.6 mg/L), accounting for 7.7% of the total fatty acids (TFAs) (Fig. 4D). However, despite this improvement, nervonic acid content still lower than previous study, which is about 10% and 23%, respectively (Fillet et al. 2017; Wang et al. 2023). Further systematic study is needed to achieve comparable nervonic acid content when compared to previous work.

### Elongation *KCS* gene copy number adjustment increased nervonic acid production in *Y. lipolytica*

**Fig. 5:**
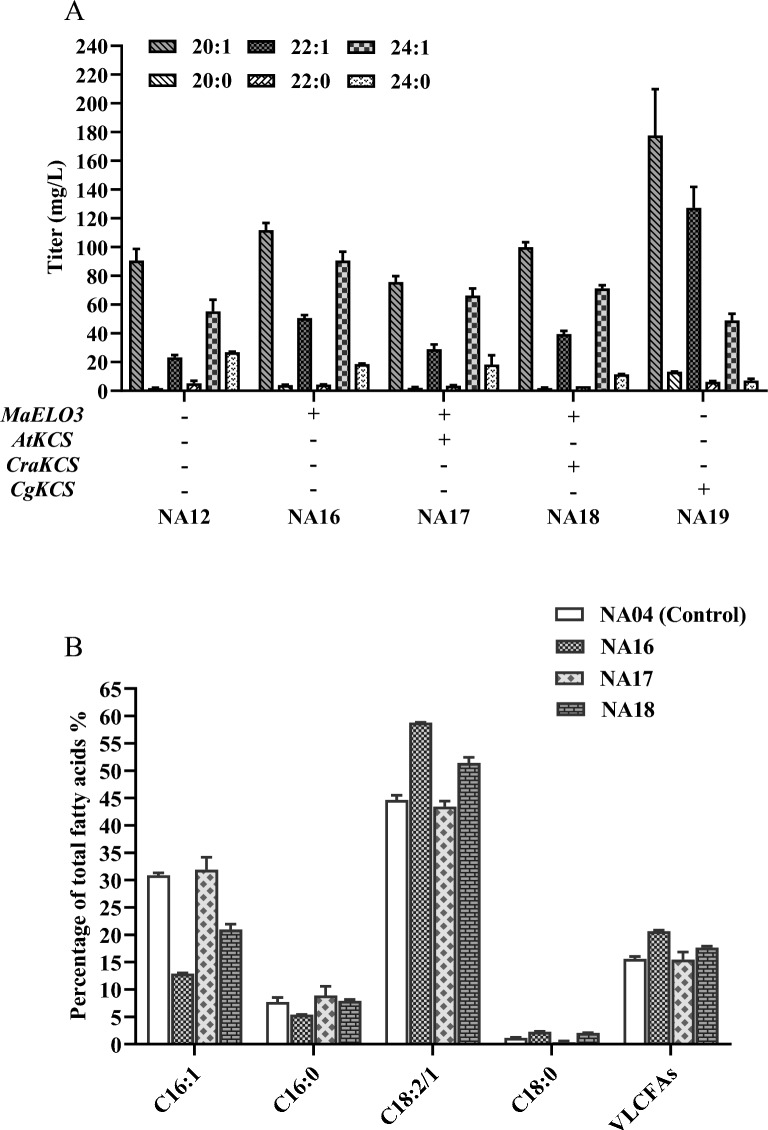
Improvement of nervonic acid production in *Y. lipolytica* through elongation *kcs* gene copy number adjustment. **A** An extra copy of *KCS* gene was overexpressed in NA12. *MaELO3*: *Mortierella alpina ELO3* gene; *AtKCS*: *Arabidopsis thaliana KCS* gene; *CgKCS*: *Cardamine graeca KCS* gene; *CraKCS*: *Crambe abyssinica KCS* gene. **B** Percentage of FA distribution in the engineered *Y. lipolytica* strains NA04, NA16-18. The data are the averages of two biological replicates with error bars representing standard deviations

To further develop a high-level nervonic acid production strain, we evaluated the impact of elongation genes on nervonic acid yield. For this purpose, we added an extra copy of four elongation genes *MaELO3*, *CraKCS*, *AtKCS,* and *CgKCS* by integrated plasmid pINA1269 to the strain NA12. As shown in Fig. 5A, only the extra copy of *MaELO3* enhanced the production of nervonic acid, the yield of nervonic acid increased by 63.9% and reached 90.6 mg/L. Meanwhile, the production of fatty acids C20:1 and C22:1 were significantly improved in the strain with extra copy of *CgKCS*. Since previous reports showed that increasing the copy number of *CgKCS* could boost the concentration of nervonic acid in *R. toruloides* (Fillet et al. 2017), the inconsistent results might be caused by different genetic background of the stains. This phenomenon is also not consistent with previous report about nervonic acid production in *Y. lipolytica*, the reason for the difference might due to different combination *KCSs* we selected between our research (Wang et al. 2023).

### Effect of oily substrates as auxiliary carbon sources for nervonic acid production by the engineered *Y. lipolytica*

**Fig. 6:**
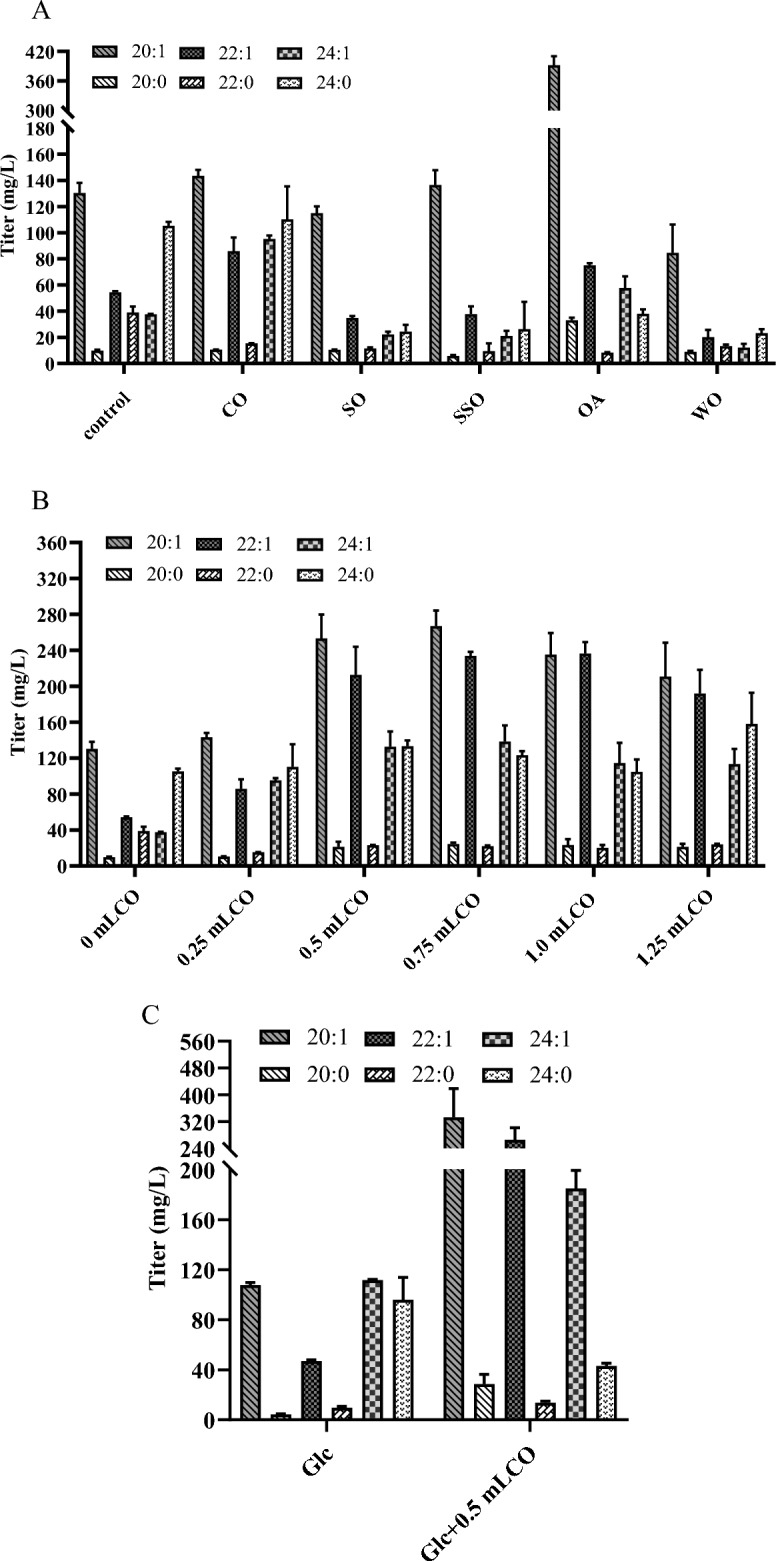
Effect of oil/oleic acid addition on nervonic acid production in engineered *Y. lipolytica*. **A** Adding different auxiliary carbon sources on nervonic acid production. **B** Colleseed oil supplemental level on the yield of nervonic acid. **C** Fermentation of strains NA15 in the YPD medium with 0.5 mL colleseed oil. The data are the averages of two biological replicates with error bars representing standard deviations

As an oleaginous yeast, *Y. lipolytica* can quickly grow to high density with a high lipid content and utilize a large number of renewable substrates and inexpensive materials such as hydrophobic substrates, crude glycerol, and lignocellulosic biomass as carbon sources (Ledesma-Amaro and Nicaud 2016; Nambou et al. 2014; Poli et al. 2014). In order to screen the most suitable carbon source for the production of nervonic acid by *Y. lipolytica*, an auxiliary carbon sources such as colleseed oil, soybean oil, sunflower seed oil, waste cooking oil, or oleic acid were supplemented to YPD medium (Fig. 6A). In the auxiliary carbon sources screening experiment, the strain NA02 was first used as the fermentation strain, and 0.25 mL of the auxiliary carbon source was added into the 50 mL YPD medium. As shown in Fig. 6A, the culture with colleseed oil as auxiliary carbon showed the highest nervonic acid productivity. In the medium supplemented with colleseed oil, the yield of nervonic acid in strain NA09 reached 132.6 mg/L, which was 2.5-fold higher than that of control YPD medium. The effect of colleseed oil concentration, on nervonic acid production was evaluated by adding 0.25 to 1.25 mL of colleseed oil to shake flask (Fig. 6B). The fermentation results showed that the highest yield of nervonic acid, 132.6 mg/L and 138.4 mg/L, was obtained when colleseed oil was added at 0.5 mL and 0.75 mL, respectively, and this yield was about 3.6-fold higher than that of the medium without colleseed oil. Considering the cost efficiency, for the following experiments we chose to add 0.5 mL colleseed oil into 50 mL fermentation medium.

In order to investigate the reason why colleseed oil was the most suitable auxiliary carbon source for nervonic acid production in this study, the VLCFAs profile of colleseed oil was analyzed. For colleseed oil used here, C20:1 and C22:1 were the most abundant portion of the VLCFAs, little amount of C24:1 and C24:0 were observed. *Y. lipolytica* GQY-∆PEX10 was the strain that only deleted *PEX10*, a gene encoding a major peroxisomal matrix protein, from *Y. lipolytica* Po1f. When this strain was cultured in YPD supplemented with colleseed oil, its profile of VLCFA was similar to that of colleseed oil due to the absence of any modification of elongation and desaturation. After metabolic engineering of the strain, the carbon flux was significantly drained toward VLCFA, which demonstrated the success of our strategies to generate high nervonic acid production (Additional file 1: Fig. S4).

Finally, the performance of the best nervonic acid-producing strain NA15 in this study was assessed in YPD medium with or without colleseed oil. After 3 days fermentation, the yield of nervonic acid in the medium with the addition of colleseed oil was 185.0 mg/L, which was about 1.6-fold higher than that in the medium without the addition of colleseed oil, and it was the highest yield of nervonic in this study (Fig. 6C)*.*

Despite complex multistep engineering efforts, production titers in this study still lower than those of previous report (Fillet et al. 2017; Wang et al. 2023). However, the systematic engineering strategies of *Y. lipolytica* introduced in this study may provide a deep understanding of the biosynthesis of nervonic acid and other VLCFAs. It should be noted that further improvements of nervonic acid production in *Y. lipolytica* will be expected using higher biomass and lipid concentrations and controlled bioreactor.

## Conclusion

In summary, we engineered the oleaginous yeast *Y. lipolytica* following multi-level strategies for efficient accumulation of nervonic acid production. Specifically, we reconstructed the elongation pathway as well as desaturation pathway and optimized the key genes expression in fatty acid metabolism through adding gene copy and protein fusion. Furthermore, we first demonstrated that supplementing the colleseed oil as auxiliary carbon benefited the nervonic acid production. The yeast engineering strategy of pathway assembling presented in this study may be employed to optimize microbial production of other valuable VLCFAs chemistry.

### Supplementary Information

**Additional file 1: Table S1.** Codon-optimized sequences of genes used in this study. **Table S2.** Plasmids used in this study. **Table S3.** Primers used in this study. **Fig. S1.** Effects of overexpression of genes *OLE1* for the biosynthesis of nervonic acid in *Y. lipolytica.* **Fig. S2.** Genes involved in acetyl-CoA biosynthesis were overexpressed individually or in combination using hp4d promoter in the background strain NA04. The data are the averages of two biological replicates with error bars representing standard deviations. **Fig. S3.** Effects of gene knockout *MFE* and overexpression *FAA1* on neuronic acid production in *Y. lipolytica*. The data are the averages of 2 biological replicates with error bars representing standard deviations. **Fig. S4.** (A) Percentage of fatty acids in colleseed oil. (B) Fermentation of strains GQ05 and NA09 in the YPD medium with 0.5 mL colleseed oil.

## Data Availability

All data generated or analyzed during this study are included in this article.
