# Supplementary material for: De novo synthesis of nervonic acid and optimization of metabolic regulation by Yarrowia lipolytica
Source: Bioresour Bioprocess. 2023 Oct 6;10(1):70. doi: 10.1186/s40643-023-00689-6 (PMC10992393; doi:10.1186/s40643-023-00689-6)
Supplement: Supplementary file 1 — Additional file 1: Table S1. Codon-optimized sequences of genes used in this study. Table S2. Plasmids used in this study. Table S3. Primers used in this study. Fig. S1. Effects of overexpression of genes OLE1 for the biosynthesis of nervonic acid in Y. lipolytica. Fig. S2. Genes involved in acetyl-CoA biosynthesis were overexpressed individually or in combination using hp4d promoter in the background strain NA04. The data are the averages of two biological replicates with error bars representing standard deviations. Fig. S3. Effects of gene knockout MFE and overexpression FAA1 on neuronic acid production in Y. lipolytica. The data are the averages of 2 biological replicates with error bars representing standard deviations. Fig. S4. (A) Percentage of fatty acids in colleseed oil. (B) Fermentation of strains GQ05 and NA09 in the YPD medium with 0.5 mL colleseed oil. [file 40643_2023_689_MOESM1_ESM.docx]

Additional Files

**Table S1. Codon optimized sequences of genes used in this study.**

| Name | Codon optimized sequence (5’ 🡪 3’) |
| --- | --- |
| *MaELO3* | ATGGAGTCTGGCCCCATGCCCGCCGGTATCCCCTTCCCTGAGTACTACGACTTCTTCATGGACTGGAAGACCCCCCTGGCCATTGCCGCCACCTACACCGCCGCCGTCGGTCTTTTCAACCCCAAGGTGGGCAAGGTGTCCCGAGTCGTCGCCAAGTCTGCCAACGCCAAGCCCGCCGAGCGAACCCAGTCCGGTGCCGCTATGACCGCCTTCGTGTTCGTGCACAACCTGATTCTGTGCGTCTACTCTGGTATCACCTTCTACTACATGTTCCCCGCCATGGTCAAGAACTTCCGAACCCACACCCTGCACGAGGCCTACTGTGACACCGACCAGTCCCTGTGGAACAACGCCCTGGGCTACTGGGGCTACCTGTTCTACCTGTCCAAGTTCTACGAGGTCATCGACACCATCATTATCATCCTGAAGGGTCGACGATCCTCCCTGCTGCAGACCTACCACCACGCCGGTGCCATGATTACCATGTGGTCTGGCATCAACTACCAGGCCACCCCCATTTGGATCTTCGTCGTCTTCAACTCTTTCATTCACACCATCATGTACTGTTACTACGCCTTCACCTCCATTGGTTTCCACCCCCCCGGTAAAAAGTACCTGACCTCTATGCAGATTACCCAGTTCCTGGTGGGTATCACCATCGCCGTCTCCTACCTGTTCGTCCCCGGCTGCATTCGAACCCCCGGTGCCCAGATGGCCGTCTGGATCAACGTCGGTTACCTGTTCCCCCTGACCTACCTGTTCGTGGACTTCGCCAAGCGAACCTACTCTAAGCGATCTGCCATCGCCGCCCAGAAGAAGGCCCAGTAA |
| *AtKCS* | ATGACCTCCGTGAACGTCAAGCTGCTGTACCGATACGTCCTGACCAACTTCTTCAACCTGTGCCTGTTCCCCCTGACCGCCTTCCTGGCCGGCAAGGCTTCCCGACTGACCATCAACGACCTGCACAACTTCCTGTCCTACCTCCAGCACAACCTCATCACCGTCACCCTCCTGTTCGCCTTCACCGTCTTCGGCCTCGTCCTCTACATCGTCACCCGACCCAACCCCGTCTACCTTGTCGACTACTCCTGCTACCTGCCCCCCCCCCACCTCAAGGTCTCCGTCTCCAAGGTCATGGACATCTTCTACCAGATCCGAAAGGCCGACACCTCCTCCCGAAACGTCGCCTGCGACGACCCCTCCTCCCTGGACTTCCTCCGAAAGATCCAGGAGCGATCCGGCCTCGGCGATGAGACCTACTCCCCCGAGGGCCTGATCCACGTTCCCCCCCGAAAGACCTTCGCCGCCTCCCGAGAGGAGACCGAGAAGGTCATCATCGGAGCCCTCGAGAACCTGTTTGAGAACACCAAGGTCAACCCCCGAGAGATCGGCATCCTCGTCGTCAACTCCTCCATGTTCAACCCCACCCCCTCCCTGTCCGCTATGGTCGTCAACACTTTTAAGCTGCGATCCAACATCAAGTCCTTCAACCTCGGCGGCATGGGCTGTTCCGCCGGCGTTATCGCCATCGACCTCGCCAAGGACCTCCTCCACGTCCACAAGAACACCTACGCCCTGGTCGTCTCCACCGAGAACATCACCCAGGGCATCTACGCTGGCGAGAACCGATCCATGATGGTCTCCAACTGCCTCTTTCGAGTTGGCGGCGCCGCCATCCTGCTGTCCAACAAGTCCGGCGACCGACGACGAAGCAAGTACAAGCTGGTCCACACTGTGCGAACCCACACCGGCGCCGATGACAAGTCCTTCCGATGCGTCCAGCAGGAGGATGACGAGTCCGGCAAGATCGGCGTGTGCCTGAGCAAGGACATCACCAACGTCGCCGGCACCACTCTGACCAAGAACATCGCCACCCTGGGCCCCCTTATTCTGCCTCTCTCCGAGAAGTTTCTGTTCTTCGCCACCTTTGTCGCCAAGAAGCTGCTGAAGGACAAGATCAAGCATTACTACGTCCCCGACTTCAAGCTTGCCGTCGATCACTTCTGCATCCACGCCGGCGGCCGAGCCGTCATCGACGAGCTTGAGAAGAACCTGGGACTCTCCCCCATCGACGTCGAGGCCTCCCGATCCACCCTGCACCGATTCGGCAACACCTCTTCCTCCTCCATCTGGTACGAGCTTGCCTACATCGAGGCCAAGGGACGAATGAAGAAGGGCAACAAGGCCTGGCAGATCGCCCTCGGCTCCGGCTTCAAGTGCAACTCGGCCGTCTGGGTCGCCCTGCGAAACGTCAAGGCCTCCGCCAACTCCCCCTGGCAGCACTGCATCGACCGATACCCCGTTAAGATCGACTCCGACCTGTCCAAGTCCAAGACCCACGTCCAGAACGGCCGATCCTAA |
| *CraKCS* | ATGACCTCTATCAACGTGAAGCTGCTGTACCACTACGTGATCACCAACCTGTTCAACCTGTGCTTCTTCCCTCTGACCGCCATCGTGGCCGGCAAGGCCTCTCGACTGACCATCGACGACCTGCACCACCTGTACTACTCTTACCTGCAGCACAACGTGATTACCATTGCTCCTCTGTTCGCCTTCACCGTGTTCGGCTCTATCCTGTACATCGTGACCCGACCTAAGCCTGTGTACCTGGTCGAGTACTCTTGCTACCTGCCTCCTACTCAGTGCCGATCTTCTATCTCTAAGGTGATGGACATCTTCTACCAGGTGCGAAAGGCTGACCCCTTCCGAAACGGAACCTGCGACGACTCTTCTTGGCTGGACTTCCTGCGAAAGATCCAAGAGCGATCTGGCCTGGGCGACGAGACTCACGGCCCCGAGGGCCTGCTCCAGGTGCCTCCTCGAAAGACCTTCGCCGCTGCTCGAGAAGAGACTGAGCAGGTCATCGTGGGCGCCCTGAAGAACCTGTTCGAGAACACCAAGGTGAACCCCAAGGACATCGGCATCCTGGTGGTGAACTCTTCTATGTTCAACCCCACTCCTTCTCTGTCTGCCATGGTGGTCAACACCTTCAAGCTGCGATCTAACGTGCGATCTTTCAACCTCGGCGGCATGGGCTGCTCTGCCGGCGTGATCGCCATCGACCTGGCCAAGGACCTGCTGCACGTCCACAAGAACACCTACGCTCTGGTGGTGTCTACCGAGAACATCACCTACAACATCTACGCCGGCGACAACCGATCTATGATGGTGTCTAACTGCCTGTTCCGAGTCGGCGGAGCCGCCATCCTGCTGTCTAACAAGCCCCGAGATCGACGACGATCTAAGTACGAGCTGGTGCACACCGTGCGAACCCACACCGGCGCTGACGACAAGTCTTTCCGATGCGTCCAGCAGGGCGACGACGAGAACGGCAAGACCGGCGTGTCTCTGTCTAAGGACATCACCGAGGTGGCCGGACGAACCGTGAAGAAGAACATTGCCACTCTGGGACCCCTGATTCTGCCCCTGTCTGAGAAGCTCCTGTTCTTCGTGACCTTCATGGCCAAGAAGCTGTTCAAGGACAAGGTGAAGCACTACTACGTGCCCGACTTTAAGCTGGCTATCGACCACTTCTGCATCCACGCTGGCGGCCGAGCCGTGATCGACGTGCTGGAAAAGAACCTGGGACTCGCTCCCATTGACGTCGAGGCTTCTCGATCTACCCTGCACCGATTCGGCAACACCTCTTCTTCGTCTATCTGGTACGAACTGGCCTACATCGAGGCCAAGGGCCGAATGAAGAAGGGCAACAAGGTCTGGCAGATCGCCCTCGGCTCTGGCTTCAAGTGCAACTCTGCCGTGTGGGTCGCCCTGTCTAACGTGAAGGCCTCTACCAACTCTCCCTGGGAGCACTGCATTGATCGATACCCCGTGAAGATCGACTCTGACTCTGCCAAGTCTGAGACTCGAGCCCAGAACGGCCGATCTTAA |
| *CgKCS* | GCGCGCATGACCTCTATCAACGTGAAGCTGCTGTACCACTACGTCCTGACCAACTTCTTCAACCTGTGTCTGTTCCCCCTGACCGCTTTCCCTGCTGGCAAGGCTTCTCAGCTGACCACCAACGACCTGCACCACCTGTACTCCTACCTGCACCACAACCTGATTACCGTGACCCTGCTGTTCGCCTTCACCGTCTTCGGTTCTATCCTGTACATTGTGACCCGACCCAAGCCCGTGTACCTGGTCGACTACTCCTGTTACCTGCCTCCCCGACACCTGTCTTGCGGAATCTCCCGAGTCATGGAGATCTTCTACGAGATTCGAAAGTCTGACCCTTCCCGAGAGGTGCCTTTCGACGACCCCTCTTCCCTGGAGTTCCTGCGAAAGATTCAGGAGCGATCTGGTCTGGGAGACGAGACCTACGGACCTCAGGGTCTGGTGCACGACATGCCCCTGCGAATGAACTTCGCTGCTGCCCGAGAGGAGACCGAGCAGGTCATCAACGGAGCTCTGGAGAAGCTGTTCGAGAACACCAAGGTGAACCCCCGAGAGATCGGTATTCTGGTGGTCAACTCTTCCATGTTCAACCCCACCCCCTCTCTGTCCGCCATGGTGGTCAACACCTTCAAGCTGCGATCTAACATCAAGTCTTTCTCCCTGGGCGGAATGGGTTGTTCCGCCGGCATCATTGCTATTGACCTGGCCAAGGACCTGCTGCACGTCCACAAGAACACCTACGCTCTGGTGGTCTCTACCGAGAACATCACCCACTCCACCTACACCGGCGACAACCGATCTATGATGGTGTCCAACTGCCTGTTCCGAATGGGTGGCGCTGCCATTCTGCTGTCTAACAAGGCCGGCGACCGACGACGATCCAAGTACAAGCTGGCTCACACCGTGCGAACCCACACCGGAGCTGACGACCAGTCTTTCCGATGTGTCCGACAGGAAGACGACGACCGAGGCAAGATCGGAGTGTGCCTGTCCAAGGACATTACCGCTGTGGCCGGCAAGACCGTCACCAAGAACATTGCTACCCTGGGACCTCTGGTCCTGCCTCTGTCTGAGAAGTTCCTGTACGTGGTCTCCCTGATGGCTAAGAAGCTGTTCAAGAACAAGATCAAGCACACCTACGTGCCCGACTTCAAGCTGGCCATCGACCACTTCTGTATTCACGCTGGAGGTCGAGCCGTGATTGACGTCCTGGAGAAGAACCTGGCTCTGTCTCCCGTGGACGTCGAGGCCTCTCGATCCACCCTGCACCGATTCGGAAACACCTCTTCCTCTTCCATCTGGTACGAGCTGGCTTACATTGAGGCCAAGGGACGAATGAAGAAGGGTAACAAGGTCTGGCAGATCGCTATTGGTTCTGGCTTCAAGTGTAACTCCGCTGTGTGGGTCGCCCTGTGCAACGTGAAGCCCTCTGTCAACTCCCCCTGGGAGCACTGCATCGACCGATACCCCGTGGAGATTAACTACGGTTCTTCCAAGTCTGAGACCCGAGCCCAGAACGGACGATCCTAGGCTAGC |
| *MaD15D* | GCCCCTCCCCACGTCGTGGACGAGCAGGTGCGACGACGAATCGTGGTCGAGGACGAGATTCAGTCTAAGAAGCAGTTCGAGCGAAACTACGTCCCCATGGACTTCACCATCAAGGAGATTCGAGACGCCATCCCCGCTCACCTGTTCATTCGAGACACCACCAAGTCTATCCTGCACGTCGTCAAGGACCTGGTGACCATCGCCATTGTCTTCTACTGTGCTACCTTCATCGAGACCCTGCCTTCCCTGGCTCTGCGAGTGCCTGCTTGGATTACCTACTGGATCATTCAGGGAACCGTGATGGTCGGTCCCTGGATTCTGGCCCACGAGTGCGGCCACGGAGCTTTCTCTGACTCCAAGACCATCAACACCATTTTCGGATGGGTGCTGCACTCGGCTCTGCTGGTCCCTTACCAGGCTTGGGCTATGTCTCACTCCAAGCACCACAAGGGTACCGGCTCTATGTCCAAGGACGTGGTCTTCATCCCCGCTACCCGATCTTACAAGGGTCTGCCTCCCCTGGAGAAGCCTGCTGCTGAGGAAGAGGTGCTGGAGCAGGAGCATCATCACCACGAGGAGTCCATCTTCGCCGAGACCCCCATCTACACCCTGGGAGCTCTGTTCTTCGTGCTGACCCTGGGTTGGCCCCTGTACCTGATCATGAACTTCTCTGGTCACGAGGCTCCCCACTGGGTGAACCACTTCCAGACCGTGGCTCCCCTGTACGAGCCTCACCAGCGAAAGAACATTTTCTACTCTAACTGTGGCATCGTCGCCATGGGCTCTATCCTGACCTACCTGTCTATGGTGTTCTCTCCCCTGACCGTCTTCATGTACTACGGTATCCCCTACCTGGGCGTGAACGCTTGGATCGTCTGCATTACCTACCTGCAGCACACCGACCCCAAGGTGCCCCACTTCCGAGACAACGAGTGGAACTTCCAGCGAGGCGCCGCTTGTACCATCGACCGATCCTTCGGAACCATTGTCAACCACCTGCACCACCACATTGGAGACTCTCACCAGTGCCACCACATGTTCTCCCAGATGCCCTTCTACAACGCCGTGGAGGCTACCAAGCACCTGAAGGCCAAGCTGGGCAAGTACTACATCTTCGACGACACCCCCATTGCCAAGGCTCTGTACCGAAACTGGCGAGAGTGTAAGTTCGTGGAGGACGAGGGCGACGTGGTCTTCTACAAGCAC |
| *CsD15D* | ATGACCGAGTCCCACGCCTCTGAGGAGATGGCTCGAGAGGAGAAGGGAGACTACCCCATTAAGGTGGCCAACGGCATCCGAAACCAGAACGGAGACTTCGACCTGTCTGACCCTCCTCCCTTCAAGATCGCTGAGATTCGAGCCGCTATTCCCAAGCACTGTTGGGTGAAGAACCCCTGGCGATCTCTGTCCTACGTCTTCCGAGACCTGTTCATCATTTTCGCCCTGGCTTTCGCCGCTTTCTACTCTGACACCTGGGTGGTCTGGCCCTTCTACTGGGCCGCTCAGGGTACCATGTTCTGGGCTCTGTTCGTCCTGGGTCACGACTGTGGTCACGGCTCTTTCTCCAACTCTCCCGAGCTGAACTCCGCCGTGGGACACATTCTGCACTCTGCTATCCTGGTCCCCTACAACGGTTGGCGAATCTCCCACCGAACCCACCACCAGAACCACGGCCACGTCGAGAACGACGAGTCTTGGGTCCCCCTGACCGAGAAGATGTACAAGCAGCTGGACGAGAAGACCAAGCGACTGCGATTCAAGGTGCCCTTCCCCCTGTTCGCCTACCCCTTCTACCTGTGGAACCGATCCCCCGGCAAGGAAGGCTCTCACTTCAACCCCTACTCCAAGCTGTTCACCCCCTCTGAGCGAAACCAGATCATTACCTCCACCGTCTGTTGGTCTACCATGGCCGCTCTGCTGGTGTGCCTGTCCTTCATTGTCGGACCCGTGCAGGTCCTGATGCTGTACGTGGTCCCCTACTGGATTTTCGTGATGTGGCTGGACATCGTCACCTACCTGCACCACCACGGATACGAGCAGAAGCTGCCCTGGTACCGAGGCAAGGAGTGGTCTTACCTGCGAGGCGGACTGACCACCGTGGACCGAGACTACGGCATCTTCAACAACATTCACCACGACATCGGAACCCACGTCATTCACCACCTGTTCCCCCAGATCCCCCACTACCACCTGGTGGAGGCTACCAAGGCTGCTAAGCCTGTCCTGGGCAAGTACTACCGAGAGCCCCGAAAGTCCGGCCCCATTCCCGTGCACCTGATCGAGAACCTGGTCAAGTCCATTTCTCAGGACCACTACGTGTCTGACAACGGAGAGGTGGTCTACTACCAGACCGACCCCGAGCTGAACAACAACAACAACAAGAAGATCTCCGAGGCTAAGCAGATGTAG |

**Table S2. Plasmids used in this study**

| Plasmids | Descriptions | Source |
| --- | --- | --- |
| pINA1269 | *Y. lipolytica* integrative plasmid, hp4d promoter, *XPR2* terminator, *LEU2* selection marker, Amp^r^ | Madzak et al.^1^ |
| pINA1312 | *Y. lipolytica* integrative plasmid, hp4d promoter, *XPR2* terminator, *ura3d1* selection marker, Km^r^ | Madzak et al.^1^ |
| pCRISPRyl_A3 | pCRISPRyl with A3 targeting sgRNA | Zhang et al.^2^ |
| pHR- A3_hrGFP | 1kb_ A3_up-UAS8B-TEF-hrGFP-CYC-1kb_F1_down, CEN1 URA3 AmpR ColE1 | Zhang et al.^2^ |
| pHR- A3-*AtKCS* | *AtKCS* replace hrGFP in pHR-A3_hrGFP | Gao et al.^3^ |
| pCRISPRyl_F1 | pCRISPRyl with F1 targeting sgRNA | Zhang et al.^2^ |
| pHR- F1_hrGFP | 1kb_ F1_up-UAS8B-TEF-hrGFP-CYC-1kb_F1_down, CEN1 URA3 AmpR ColE1 | Zhang et al.^2^ |
| pHR- F1*-MaELO3* | *MaELO3* replace hrGFP in pHR-F1 _hrGFP | Gao et al.^3^ |
| pCRISPRyl_F1-3 | pCRISPRyl with F1-3 targeting sgRNA | Zhang et al.^2^ |
| pHR- F1-3_hrGFP | 1kb_ F1-3_up-UAS8B-TEF-hrGFP-CYC-1kb_F1_down, CEN1 URA3 AmpR ColE1 | Zhang et al.^2^ |
| pHR- F1-3-*CraKCS* | *CraKCS* replace hrGFP in pHR-F1-3 _hrGFP | This study |
| pHR_A1-2_hrGFP | 1kb_A1-2_up-UAS8B-TEF-hrGFP-CYC-1kb_F1_down, CEN1 URA3 AmpR ColE1 | Zhang et al.^2^ |
| pCRISPRyl_A1-2 | pCRISPRyl with A1-2 targeting sgRNA | Zhang et al.^2^ |
| p1312-UT- *CsD15D* | p1312-UT constitutively expressed codon optimized *CsD15D* | This study |
| p1312-UT- *MaD15D* | p1312-UT constitutively expressed codon optimized *MaD15D* | This study |
| pHR_E1-3_CgKCS-L-MaD15D | *CgKCS-L-MaD15D* replace hrGFP in pHR-E1-3_hrGFP | This study |
| pHR_E1-3_hrGFP | 1kb_E1-3_up-UAS8B-TEF-hrGFP-CYC-1kb_F1_down, CEN1 URA3 AmpR ColE1 | Zhang et al.^2^ |
| pCRISPRyl_E1-3 | pCRISPRyl with E1-3 targeting sgRNA | Zhang et al.^2^ |
| pINA1312-P_UT_-OLE1 | pINA1312-P_UT_ vector containing truncated version of *OLE1* gene | This study |
| pINA1312-P_UT_-OLE1-L-DGA1 | pINA1312-P_UT_ vector containing truncated version of *OLE1* and *DGA1* gene | This study |
| pINA1269-OLE1-L-DGA1 | pINA1269- vector containing truncated version of *OLE1* and *DGA1* gene | This study |
| pINA1312-P_UT_-DGA1-L-OLE1 | pINA1312-P_UT_ vector containing truncated version of *OLE1* and *DGA1* gene | This study |
| pINA1269-DGA1 | pINA1269 vector containing truncated version of *DGA1* gene | This study |
| pINA1269-P_UT_-MaELO3 | pINA1269-P_UT_ vector containing truncated version of *MaELO3*gene | This study |
| pINA1269-P_UT_-CgKCS | pINA1269-P_UT_ vector containing truncated version of *CgKCS* gene | This study |
| pINA1269-P_UT_-MaELO3*-*AtKCS | pINA1269-P_UT_-MaELO3 vector containing truncated version of *AtKCS* gene | This study |
| pINA1269-P_UT_-MaELO3-CraKCS | pINA1269-P_UT_-MaELO3 vector containing truncated version of *CraKCS* gene | This study |
| p1269ACL | pINA1269 vector containing *ACL* gene | This study |
| p1269ACC1 | pINA1269 vector containing *ACC1* gene | This study |
| p1269ACS2 | pINA1269 vector containing *ACS2* gene | This study |
| p1269ACL-ACS2 | pINA1269 vector containing *ACL* and *ACS2* genes | This study |

**Table S3. Primers used in this study**

| Primer name | Sequences |
| --- | --- |
| 32UTAtKCS-f | gagtataagaatcattcaaacacgtgatgacctccgtgaacgt |
| 32UTAtKCS-r | tggggacaggccatggaggtaccggatccttaggatcggccgt |
| 32UTCraKCS-f | gtataagaatcattcaaacacgtgatgacctctatcaacgtgaagct |
| 32UTCraKCS-r | caggccatggaggtaccggatccttaagatcggccgttctgggct |
| 32UTCgKCS-f | caagtgagatgcccgtgtccgctagcttatcgatacgcg |
| 32UTCgKCS-r | gagtgttacacatggaattcggacacgggcatctcacttgcgtatg |
| 32UTMaELO3-f | gtataagaatcattcaaacacgtgatggagtctggccccatgcccgc |
| 32UTMaELO3-r | aggccatggaggtacc ggatcc ttactgggccttcttctgggc |
| F1_MaELO3-f | gagaatacaacgcctgccat actagt aagctagcttatcgatacgcgt |
| F1_MaELO3-r | caagcctgtggaggacttcaag cctagg ggacacgggcatctcact |
| A3_AtKCS-f | ccttctgagtataagaatcattcaaaggcgcgccatggtgagcaagcagat |
| A3_AtKCS-r | gtaagcgtgacataactaattacatgaggctagcttacacccactcgtgcag |
| F1-3_CraKCS-f | gtataagaatcattcaaaggcgcgccatgacctctatcaacgtgaag |
| F1-3_CraKCS-r | cataactaattacatgaggctagcttaagatcggccgttctg |
| AXP_CgKCS-f | cttctgagtataagaatcattctaaggcgcgcatgacctctatcaacgtg |
| AXP_CgKCS-r | actaattacatgaggctaggctagcctaggatcgtccgttctgggc |
| A1-2_CgKCS-L-MaD15D-f | ccttctgagtataagaatcagcgcgcatgacctctatcaa |
| A1-2_CgKCS-L-MaD15D-r | aattacatgaggctagcttacactagtgcttgtagaagac |
| E1-3_CgKCS-L-MaD15D-f | gtataagaatcattcaaaggcgcgccggcgcgcatgacctctatcaacg |
| E1-3_CgKCS-L-MaD15D-r | atgtaagcgtgacataactaattacatgaggctagctagtgcttgtagaaga |
| 32UTCgKCS-L-MaD15D-f | gagcccagaacggacgatccggttctggtgcccctccccacgtcg |
| OLE1-L-DGA1-F | ataagaatcattcaaacacgatggtgaaaaacgtgga |
| OLE1-L-R | ctaagcagccatgccagaca |
| DGA1-L-F | ggctgcttagtgggttctggtatggaagtccgacgacgaaaaa |
| OLE1-L-DGA1-R | aggccatggaggtaccggatccctactggttctgcttgta |
| DGA1-L-OLE1-F | acttggtccacgtttttcaccataccagaaccctactggttctgcttgta |
| DGA1-L-OLE1-R | ctaagcagccatgccagaca |

Fig S1. Effects of overexpression of genes *OLE1* for the biosynthesis of nervonic acid in *Y. lipolytica*.

Fig S2. Genes involved in acetyl-CoA biosynthesis were overexpressed individually or in combination using hp4d promoter in the background strain NA04. The data are the averages of two biological replicates with error bars representing standard deviations.

Fig S3. Effects of gene knockout *MFE* and overexpression *FAA1* on neuronic acid production in *Y. lipolytica*. The data are the averages of 2 biological replicates with error bars representing standard deviations.

A

B

Fig S4. (A) Percentage of fatty acids in colleseed oil. (B) Fermentation of strains GQ05 and NA09 in the YPD medium with 0.5 mL colleseed oil.

**REFERENCES**

1. Madzak, C.; Gaillardin, C.; Beckerich, J. M., Heterologous protein expression and secretion in the non-conventional yeast *Yarrowia lipolytica*: a review. Journal of Biotechnology 2004, 109 (1-2), 63-81.
2. Zhang, X. K.; Wang, D. N.; Chen, J.; Liu, Z. J.; Hua, Q., Metabolic engineering of β-carotene biosynthesis in *Yarrowia lipolytica*. Biotechnology Letters 2020, 42 (1), 945-956.
3. Gao, Q.; Yang, J.-L.; Zhao, X.-R.; Liu, S.-C.; Liu, Z.-J.; Wei, L.-J.; Hua, Q., *Yarrowia lipolytica* as a metabolic engineering platform for the production of very-long-chain wax esters. Journal of Agricultural and Food Chemistry 2020, 68 (39), 10730-10740.
